# Sleeping trees and sleep-related behaviours of the siamang (*Symphalangus syndactylus*) in a tropical lowland rainforest, Sumatra, Indonesia

**DOI:** 10.1007/s10329-020-00849-8

**Published:** 2020-07-27

**Authors:** Nathan J. Harrison, Ross A. Hill, Cici Alexander, Christopher D. Marsh, Matthew G. Nowak, Abdullah Abdullah, Helen D. Slater, Amanda H. Korstjens

**Affiliations:** 1 Department of Life and Environmental Sciences, Bournemouth University, Talbot Campus, Fern Barrow, Poole, BH12 5BB UK; 2 Aarhus Institute of Advanced Studies, Aarhus University, Høegh-Guldbergs Gade 6B, 8000 Aarhus C, Denmark; 3 Sumatran Orangutan Conservation Programme, PanEco Foundation, Chileweg 5, 8415 Berg am Irchel, Switzerland; 4 Department of Anthropology, Southern Illinois University, 1000 Faner Drive, Carbondale, IL 62901 USA; 5 Department of Biology, Syiah Kuala University, Banda Aceh, Aceh 23111 Indonesia

**Keywords:** Primate, Hylobatidae, Predation, Gunung Leuser National Park, Leuser Ecosystem

## Abstract

Sleeping tree selection and related behaviours of a family group and a solitary female siamang (*Symphalangus syndactylus*) were investigated over a 5-month period in northern Sumatra, Indonesia. We performed all day follows, sleeping tree surveys and forest plot enumerations in the field. We tested whether: (1) physical characteristics of sleeping trees and the surrounding trees, together with siamang behaviours, supported selection based on predation risk and access requirements; (2) the preferences of a solitary siamang were similar to those of a family group; and (3) sleeping site locations within home ranges were indicative of home range defence, scramble competition with other groups or other species, or food requirements. Our data showed that (1) sleeping trees were tall, emergent trees with some, albeit low, connectivity to the neighbouring canopy, and that they were surrounded by other tall trees. Siamangs showed early entry into and departure from sleeping trees, and slept at the ends of branches. These results indicate that the siamangs’ choice of sleeping trees and related behaviours were strongly driven by predator avoidance. The observed regular reuse of sleeping sites, however, did not support anti-predation theory. (2) The solitary female displayed selection criteria for sleeping trees that were similar to those of the family group, but she slept more frequently in smaller trees than the latter. (3) Siamangs selected sleeping trees to avoid neighbouring groups, monopolise resources (competition), and to be near their last feeding tree. Our findings indicate selectivity in the siamangs’ use of sleeping trees, with only a few trees in the study site being used for this purpose. Any reduction in the availability of such trees might make otherwise suitable habitat unsuitable for these highly arboreal small apes.

**Electronic supplementary material:**

The online version of this article (10.1007/s10329-020-00849-8) contains supplementary material, which is available to authorized users.

## Introduction

Studies of sleeping site selection across the Primate order have revealed a diverse range of behaviours and choice of sites. Sleeping sites are important as their suitability, abundance, and use can ultimately affect an individual’s reproductive success and impact survival rates (Cheyne et al. 2012; Lutermann et al. 2010; Phoonjampa et al. 2010). Many primate species opt for large emergent trees with big branches, or for tree holes, caves or trees near rivers for sleeping in (Phoonjampa et al. 2010; Qihai et al. 2009; Schmid 1998). Despite primates spending up to 50% of their time at their sleeping sites (Anderson 2000), sleeping trees are not always taken into consideration when habitat suitability, primate behaviours, distributions or densities are studied.

Most anthropoids are diurnal to avoid attack by nocturnal predators (Anderson 1984), and display various behaviours that help minimise the risks of detection and predation whilst sleeping at night. Behaviours exhibited around sleeping trees include selecting suitable (e.g. the tallest) trees to sleep in (Brividoro et al. 2019); entering sleeping sites before nocturnal predators become active (Anderson 1998; Reichard 1998); irregularly using the same sleeping tree (Teichroeb et al. 2012; Whitten 1982); using familiar sleeping sites with known escape routes (Di Bitetti et al. 2000; Struhsaker 1967); moving rapidly into the sleeping tree and remaining quiet near or at the sleeping site (Qihai et al. 2009); defecating away from the sleeping site, thereby reducing olfactory cues that alert predators to their presence (Fan and Jiang 2008); and moving away from the sleeping site immediately after use (Reichard 1998). Sleeping trees on which vines and lianas grow are considered to increase predation risk as felids and humans can use them to climb up into the canopy, whilst snakes can use them for concealment (Fei et al. 2012; Phoonjampa et al. 2010). Sleeping location in the tree can also be related to predation risk, as it can be argued that sleeping at the end of a branch could expose an individual to avian predators, or conversely that it may enhance the early detection of arboreal predators through branch vibrations (Fan and Jiang 2008; Fei et al. 2012). The documented killing of a juvenile siamang (*Symphalangus syndactylus*) by a clouded leopard (*Neofelis diardi*) in southern Sumatra (Morino 2010) highlights the importance of anti-predation strategies in these apes.

Primate sleeping site selection, however, is unlikely to be solely driven by predator avoidance, with variables such as distance to food (Fan and Jiang 2008), range defence (Heymann 1995), thermoregulation (Fei et al. 2019), comfort and protection from the elements and parasites (Largo 2009; Whitten 1982), also likely to be non-mutually exclusive influencing factors. Sleeping sites may be selected at the edge of the home range to facilitate range defence (Day and Elwood 1999) or nearer to the centre of the home range to avoid inter-group aggression. For example, around 20% of sleeping trees of lar gibbons (*Hylobates lar*) and pileated gibbons (*Hylobates pileatus*) in Thailand were in areas of the home range that overlapped with those of other groups (Reichard 1998; Phoonjampa et al. 2010). By contrast, Cao Vit gibbons (*Nomascus nasutus*) in China actively avoided sleeping trees in areas of home range overlap (Fei et al. 2012).

Resource availability may also drive sleeping site selection, as travel has energetic and temporal limitations (Cannon and Leighton 1994), and can lead to sleeping sites being located near important food sources (Anderson 1984; Phoonjampa et al. 2010). However, sleeping sites are usually not food trees, as shown for agile gibbons (*Hylobates agilis*) (Gittins 1982) and lar gibbons (Reichard 1998), as they may attract unwanted attention from inter- and intraspecific competitors and predators.

Hylobatids (gibbons and siamangs) are highly territorial, arboreal apes (Leighton 1987; O'Brien et al. 2003) that are known to sleep on the bare branches of tall trees that have wide trunks and crowns that emerge above the surrounding canopy (Cheyne et al. 2012; Fei et al. 2012, 2017). Opportunistic observations indicate that siamangs sleep and sing in emergent trees (O'Brien et al. 2003), although how their sleep-related behaviours and sleeping tree selection compare with those of other gibbon species has not been reported. Siamangs differ from other hylobatids by having more group members, greater group cohesion, and a greater tendency to share the same sleeping tree (Palombit 1996). Groups of up to five siamangs consisting of mated pairs and their offspring have been documented, but solitary males and females also occur (Aldrich-Blake and Chivers 1973; Chivers et al. 1975; Gittins and Raemaekers 1980; N. J. H., personal observation). Whether there are differences in habitat and sleeping tree use between groups and solitary siamangs is unknown.

In this study, we aimed to (1) determine the physical characteristics of siamang sleeping trees and the surrounding forest, and record siamang behaviours to determine whether predation risk or access requirements supported tree selection; (2) compare sleeping tree preferences of a solitary siamang with those of a family group; and (3) assess whether sleeping site locations were indicative of range defence, scramble competition or access to food resources (Table 1).

**Table 1:** Drivers and associated predictions for the investigation of sleeping trees and sleep-related behaviours of siamangs at Gunung Leuser National Park

Driver	Predictions	Method	Prediction supported?
Predation	Siamangs will use many sleeping trees, reusing them infrequently to avoid predictability to predators	Count sleeping trees used and frequency of reuse	No
	Sleeping trees will be tall, with wide trunks, high boles and large crowns to reduce access for ground predators (e.g. felids)	Measure tree height, trunk diameter, height to first branch and crown width of sleeping trees relative to those of other emergent trees in the area	Partly (not diameter)
	Sleeping trees will emerge above the surrounding canopy to reduce access for ground and tree-dwelling predators (e.g. felids, snakes)	Measure relative height of sleeping trees to that of surrounding trees/canopy height	Yes
	Sleeping trees will have few vines and lianas and relatively few branches to reduce hiding places and access routes for predators (e.g. felids, snakes)	Measure vine and liana load and count the number of small branches	Partly (branches only)
	Sleeping trees will be surrounded by tall trees with wide trunks, high boles, and large crowns, but these will not be emergent or taller than the sleeping tree to further reduce access for ground and tree-dwelling predators (e.g. felids, snakes)	Measure tree height, trunk diameter, height to first branch and crown width of trees surrounding the sleeping trees and of other emergent trees in the area not used for sleeping in by siamangs	Partly (not diameter)
	Siamangs will move in and out of sleeping trees when nocturnal predators are least active (e.g. felids)	Record time of entry into and departure from sleeping tree relative to dusk and dawn	Yes
	Siamangs will require adequate cover and connectivity to neighbouring trees for swift access to the sleeping tree, to reduce their exposure to ground and aerial predators (e.g. raptors)	Measure canopy connectivity to neighbouring trees, and crown sizes of sleeping trees and control trees	Yes
	Siamangs will move quickly to and from the sleeping place within a sleeping tree	Record time to reach and leave the sleeping place in relation to entering and leaving the sleeping tree	No
	Siamangs will sleep above the mean forest canopy height to reduce their exposure to ground predators (e.g. felids, snakes, humans)	Compare relative height of sleeping tree to the mean height of trees/canopy surrounding the sleeping tree	Yes
	Siamangs will sleep at the ends of branches to facilitate escape from predators	Record sleeping location of individual siamangs in the sleeping tree	Yes
Access requirements	There will be adequate connectivity between the sleeping tree crown and the surrounding forest canopy for access via brachiation	Measure canopy connectivity of sleeping trees in comparison to that of similarly sized control emergent trees in the area	Yes
Group requirements	Groups of siamangs will have larger, more stable sleeping trees than a solitary siamang	Compare characteristics of sleeping trees used by a family group of siamangs with those of a solitary individual	Yes
	An individual siamang will have fewer constraints regarding sleeping tree characteristics, and will therefore use more trees and each one less frequently than a group	Compare sleeping tree usage by a family group of siamangs with that of a solitary individual	Yes
Range defence	Sleeping trees will be located towards the boundaries of home ranges to deter their use by neighbouring groups	Record locations of sleeping trees within the periphery of the home range using a Global Positioning System (GPS) unit	No
	Sleeping trees will be located in the central area of home ranges to avoid inter-group aggression	Record locations of sleeping trees within the core of the home range using a GPS unit	Yes
Competition	There will be competition for sleeping trees with other medium-bodied diurnal primate species, and also with other siamangs	Monitor usage of sleeping trees by other primate species when not occupied by siamangs	Yes
Food resources	Siamangs will sleep close to their first feeding tree of the following morning	Measure distance between sleeping tree and first feeding tree relative to average half-hourly distance	No
	Siamangs will sleep close to their last feeding tree of that evening	Measure distance between last feeding tree to next sleeping tree relative to average half-hourly distance travelled	Yes

## Methods

### Study site and subjects

This study was carried out at the Sikundur Monitoring Post, Sumatra, Indonesia. Sikundur lies within both the Gunung Leuser National Park and the wider Leuser Ecosystem, and is a degraded forest because of historic logging and its proximity to human settlements and associated forest disturbance (Priatna et al. 2006). Nevertheless, the site is one of the last remaining expanses of lowland forest in Sumatra and is home to six diurnal primate species: lar gibbons, Thomas’ leaf moneys (*Presbytis thomasi*), pig-tailed macaques (*Macaca nemestrina*), long-tailed macaques (*Macaca fascicularis*), Sumatran orangutans (*Pongo abelii*), and siamangs. Potential predators of these primates include Sumatran tigers (*Panthera tigris sondaica*), clouded leopards, pythons (*Python* spp.) and grey-headed fish eagles (*Haliaeetus ichthyaetus*).

At 30–100 m above sea level, Sikundur is lowland, and contains a series of human-made trails through dipterocarp alluvial forest (Knop et al. 2004). The site, which is close to the national park boundary, was mechanically and selectively logged on both small and large scales from the 1970s to the 1990s (de Wilde and Duyfjes 1996; Nowak 2013). Illegal logging still occurs within the protected areas, with the largest and most commercially valuable trees most frequently felled (Priatna et al. 2006). Vegetation clearance also takes place along the rivers and in other small areas for illegal plantations.

We defined a sleeping tree as one in which one or more siamangs spent a night. To maximise our understanding of siamang sleeping tree use, we followed one family group (FG) and one solitary female (SF) during the study period. FG consisted of one adult male, one adult female and their sub-adult male offspring. In June 2018, SF gave birth to an infant (undetermined sex), but because young siamangs do not leave their mother’s side until they are 3 months or older, the infant was not included in this study. The territories of FG and SF were adjacent, and FG and SF encountered each other frequently. Other siamang groups whose home ranges may have overlapped with those of FG and SF were not habituated and could not be followed. The home ranges of siamangs are reported to be 1.5–4.8 km^2^ (Gittins and Raemaekers 1980; O'Brien et al. 2003), and their mean daily travel 0.74 km/day (range = 0.2–1.7 km/day) (Gittins and Raemaekers 1980). Siamang densities at Sikundur have been estimated at 0.0–1.0 groups/km^2^ (Hankinson et al., in review), which are considered relatively low (O'Brien et al. 2003).

### Data collection

N. J. H. and Ucok Sahrizal, a local field guide, collected all the data from April to August 2018. We followed siamangs for a total of 53 days. We followed each siamang unit (FG and SF) on 3–5 consecutive days up to 11 days/month (Table 2), from when they left a sleeping tree at dawn (if known) until they entered a sleeping tree at dusk. We aimed to arrive at the sleeping tree well before sunrise (> 30 min). If the previous night’s sleeping tree was not known, we located the siamangs by searching in areas where they are known to range frequently, or by following their morning long calls. Complete-day follows occurred when siamangs were followed from sleeping tree to sleeping tree. Partial day follows occurred when the previous night’s sleeping tree was unknown, if we lost track of the group due to unfavourable terrain or vegetation, or we abandoned data collection due to adverse weather conditions (Table 2). We were less able to follow SF due to her elusive, solitary nature and because the landscapes she occupied were difficult to access. Nevertheless, we re-located siamangs in their sleeping trees on all 5 partial day follows for FG and on 9 of 14 partial day follows for SF. Furthermore, to increase our sample size for tree use frequency, but not our overall count of sleeping trees used, we visited known sleeping trees of both units in the evening to identify which sleeping trees were being used. This method also allowed us to determine whether any other species or primate groups were using the same sleeping trees when the focal siamangs were absent. These extra evening visits were made to FG sleeping trees 33 times and to SF sleeping trees 14 times. Thus, in total, there were 60 separate records of sleeping tree use for FG and 35 for SF (Table 2).

**Table 2:** Details of complete and partial day follows of the siamang family group (*FG*) and solitary female (*SF*)

Unit	Sleeping trees (*n*)	Observed use (*n*)	Observed sleeping tree use			Total time followed
			Complete day follows (*n*)	Partial day follows (*n*)	Evening visits (*n*)	
FG	4	60	22	5	33	281 h 42 min
SF	15	35	12	9^a^	14	209 h 07 min

^a^Actual number of partial day follows for SF was 14, but we were unable to relocate her on five of these occasions

On days when no siamang follows were scheduled, we assessed the physical characteristics of sleeping trees and neighbouring trees. Forest plots measuring 25 m × 25 m oriented in a north–south and east–west direction were established with the sleeping tree as the central point. We measured or estimated nine physical characteristics of sleeping trees: diameter at breast height (DBH); height; height to first major bole; crown depth (height from first major bole to tree top); crown width (in a north, south, east, and west orientation; these data were used to calculate crown area); percentage of canopy connected to adjacent tree canopy, estimated visually (canopy connectivity); percentage of tree covered by vines and lianas on both crown and trunk estimated visually; total number of branches with an estimated diameter of 10–20 cm; and total number of branches estimated to be over 20 cm in diameter. The same measurements were made on every tree with a DBH ≥ 10 cm within the 25m × 25m plot (referred to here as ‘background trees’; Table 3). Tree heights were measured with a Nikon Forestry Pro Laser Rangefinder. A total of 22 siamang sleeping trees were identified, of which three were identified by C. D. M. prior to the study (Marsh 2019). These three additional sleeping trees, used by FG, were included in all physical characteristics and spatial analyses, but were excluded from other analyses, as use of these trees was not seen during the study period.

**Table 3:** Terminologies and definitions of tree and plot types

Term	Definition
Sleeping tree	An emergent tree used by siamangs as a sleeping location during the study period (or identified immediately prior to this study)
Control tree	An emergent tree not used by siamangs as a sleeping location during the study period
Background tree	A tree with DBH > 10 cm within a 25m × 25m sleeping plot or control plot that was not identified as a sleeping tree or control tree
Sleeping plots	Forest plot (25 m × 25 m) with a sleeping tree at the centre, surrounded by background trees
Control plots	Forest plots (25 m × 25 m) with a control tree at the centre, surrounded by background trees

*DBH* Diameter at breast height

To assess any differences between emergent trees used as sleeping sites and other emergent trees in the area, we identified 21 emergent trees that were not slept in by siamangs during the study period. Eighteen of these trees were identified using unmanned aerial vehicle data obtained from Alexander et al. (2018), and three were identified visually along the human-made trails within the Sikundur monitoring system. These emergent non-sleeping trees (control trees; Table 3) were within an area that one or both siamang units occupied; control plots were defined in the same way as sleeping plots, i.e. a control tree was at the centre of a 25m × 25m control plot. The same physical measures recorded for sleeping plots were recorded for control plots for all trees with a DBH ≥ 10 cm. We wanted to determine whether the selection of a sleeping tree was based solely on the characteristics of the tree or whether it was also influenced by characteristics of the surrounding forest. Therefore we compared sleeping trees with similarly tall but non-selected trees. In addition, we compared aspects of the surrounding forest, to assess possible factors such as accessibility for the siamangs, their competitors or predators. There was no spatial overlap between any of the forest plots. Both sleeping trees and control trees were identified by their respective local Indonesian names, and to family and genus level, but were not identified to species level because this is notoriously difficult to for Sumatran forest trees.

During siamang follows behavioural data were recorded using the 5-min scan sampling technique via the Animal Observer application (version 1.0) on an Apple iPad (Caillaud 2016). Behaviours were classified into four categories—feeding, travelling, resting, and socialising—to help us to understand the influence of feeding locations on sleeping tree locations and siamang movement through the forest. Global Positioning System (GPS) locations were recorded every 30-min using a Garmin GPSMAP 64S and associated with one for the four aforementioned behaviours. The times when individual siamangs entered a sleeping tree and when they reached their final sleeping position were recorded. Sunrise and sunset times were taken from the GPS unit based on our location. Siamang sleeping positions within a tree were recorded in the evenings and categorised as close to trunk, middle of branch, or end of branch. The height of each sleeping siamang was recorded using a Nikon Rangefinder, and the horizontal distance between individuals of FG was determined by standing below them and recording the distance with a tape measure. In the mornings, the time when a siamang was first heard moving was recorded, usually before there was enough light for visible detection. Times of exiting the sleeping tree and whether individuals had moved from where they were last seen the previous evening were also recorded. The dense foliage of the tree crowns and surrounding canopy often made it difficult to be sure of the exact entry and exit times, changes in sleeping positions and locations within trees, and heights of siamangs. Sample sizes were therefore not always consistent across variables.

### Data analysis

We first compared all sleeping trees with all control trees, and all background trees in sleeping plots with all background trees in control plots for both siamang units. We then separately compared sleeping plots of each siamang unit with control plots within their respective home ranges because the selection of sleeping trees with specific characteristics depends on the availability of trees within the home range. As most of the physical tree variables did not meet the assumption of normality, Mann–Whitney *U*-tests were applied to identify any significant differences between sleeping trees and control trees, and between background trees in sleeping plots and in control plots. We also compared sleeping trees selected by FG with those selected by SF, as well as background trees in the corresponding plots of the two siamang units. As the sample size for background trees was considerably larger than for sleeping and control trees, the median values of each plot variable were used for comparisons between these categories. In this way, plots were independent from one another, but trees within plots had similar statistical values.

The expected and observed frequencies of sleeping tree use, and any preferences for the genera of the used trees were compared using χ^2^ goodness-of-fit tests. Expected frequencies were calculated by dividing the total number of recorded uses by the number of sleeping trees used. When describing the average distances or times in metres, kilometre or minutes, respectively, the mean ± 1 SD is given, unless stated otherwise. All statistical analyses were performed in R (version 3.5.1).

To test the importance of range defence, a minimum convex polygon (MCP) was calculated for both siamang units using the GPS locations recorded every 30 min for each of the day follows, with the arbitrary border representing the extent of the distribution of the unit (as an indicator of home range periphery). Kernel density estimates (KDE) at 50% and 95% were calculated to determine the core and periphery of home ranges, respectively (Cabrera et al. 2016). The distribution of feeding trees used by siamangs was also analysed from the GPS locations recorded every 30 min using the KDE method to determine the most intensely used areas (50% KDE) of feeding trees with respect to the locations of sleeping trees and home ranges. All spatial data were processed in ArcMap (version 10.5).

### Ethical note

The relevant university, local and national authorities, including the Ministry of Research, Technology and Higher Education of the Republic of Indonesia (RISTEKDIKTI; N. J. H. permit reference 2B/TKPIPA/E5/Dit.KI/II/2018; C. D. M. permit reference 12/TKPIPA/E5/Dit.KI/XII/2015 and 1065/UN11/TU/2017), Conservation of Natural Resource, Indonesia, and Taman Nasional Gunung Leuser approved the research reported in this study, which thereby adhered to the legal requirements of Indonesia. There was no physical contact with the primates in this study.

## Results

### Frequency of sleeping tree use

**Fig. 1:**
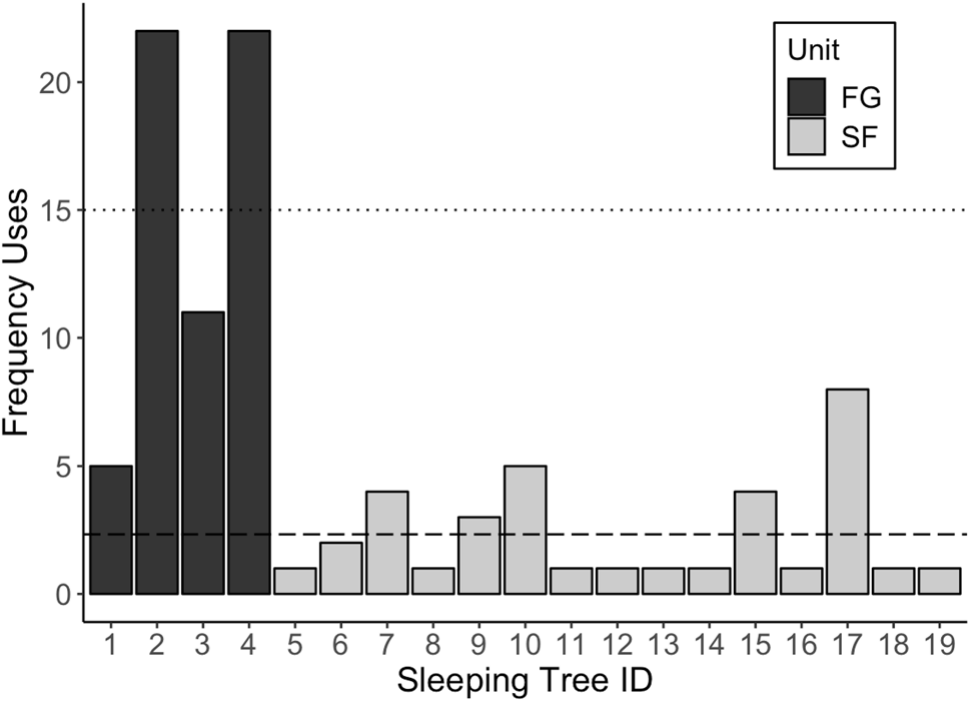
Frequency of sleeping tree use by the family group (*FG*; 60 observations) and the solitary female (*SF*; 35 observations). The* dotted line* represents the group mean level for FG if frequency was consistent (15 uses), and the* dashed line* represents the group mean level for SF if frequency was consistent (2.33 uses)

FG used four sleeping trees across 60 observations, reusing each one on five or more occasions, with significant differences in sleeping tree use (*χ*^2^ = 14.27,* df* = 3, *P* = 0.003). Two sleeping trees were used 22 times (Fig. 1). FG reused the same tree on 2 consecutive nights nine times (9/60, 15% of recorded uses). SF used a total of 15 sleeping trees across 35 observations, and also showed a significant preference for some of these trees (*χ*^2^ = 26.29,* df* = 14, *P* = 0.024), with one tree being used eight times (Fig. 1). SF reused the same sleeping tree twice on 2 consecutive nights (2/35, 5.7% of recorded uses). There was no overlap between the four sleeping trees used by FG and the 15 sleeping trees used by SF. As the sleeping locations of the two siamang units were not recorded every night, the values indicate the minimum incidence of reuse for each sleeping tree. The 19 sleeping trees used by siamangs during the study period belonged to four genera in four families. The trees used most frequently were of the genera *Intsia* (family Fabaceae; eight trees used on 68.42% of nights) and *Shorea* (family Dipterocarpaceae, eight trees used on 27.37% of nights). Two *Endospermum* trees (Euphorbiaceae; 2.11% of nights) and one *Syzygium* tree (Myrtaceae; 2.11% of nights) were also used as sleeping trees more than expected by chance (*χ*^2^ = 29.06,* df* = 3, *P* < 0.001).

### Structural characteristics of sleeping trees

A total of 43 plots were assessed (22 sleeping plots, 21 control plots) across both FG and SF home ranges. A total of 467 individual trees were measured, of which 43 were the central sleeping or control trees, with the rest classified as background trees within the plots. All sleeping and control trees were emergent above the surrounding canopy.

**Fig. 2:**
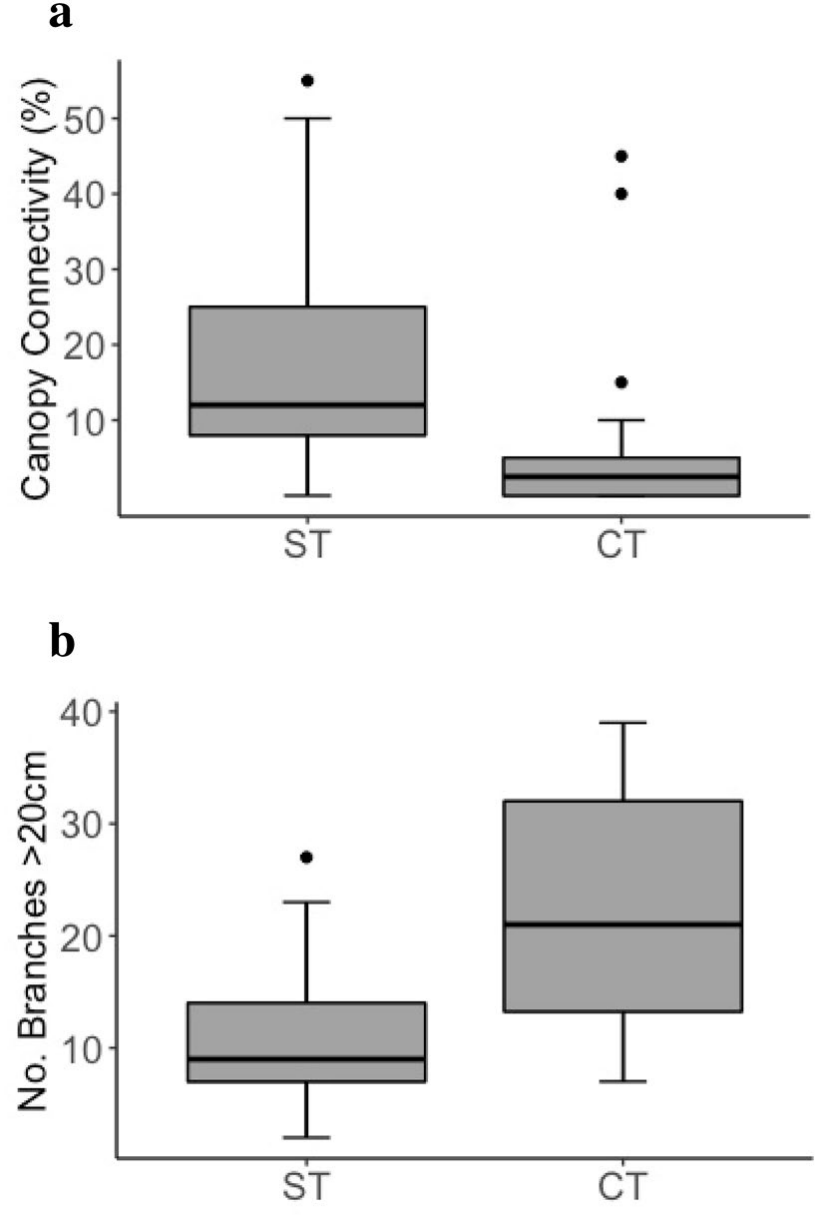
Canopy connectivity (**a**) and number of branches over 20 cm in diameter (**b**). *ST *Sleeping trees, *CT *control emergent trees.* Boxes* represent quartiles,* whiskers* are set to the 95th percentile,* dots* represent outliers

**Fig. 3:**
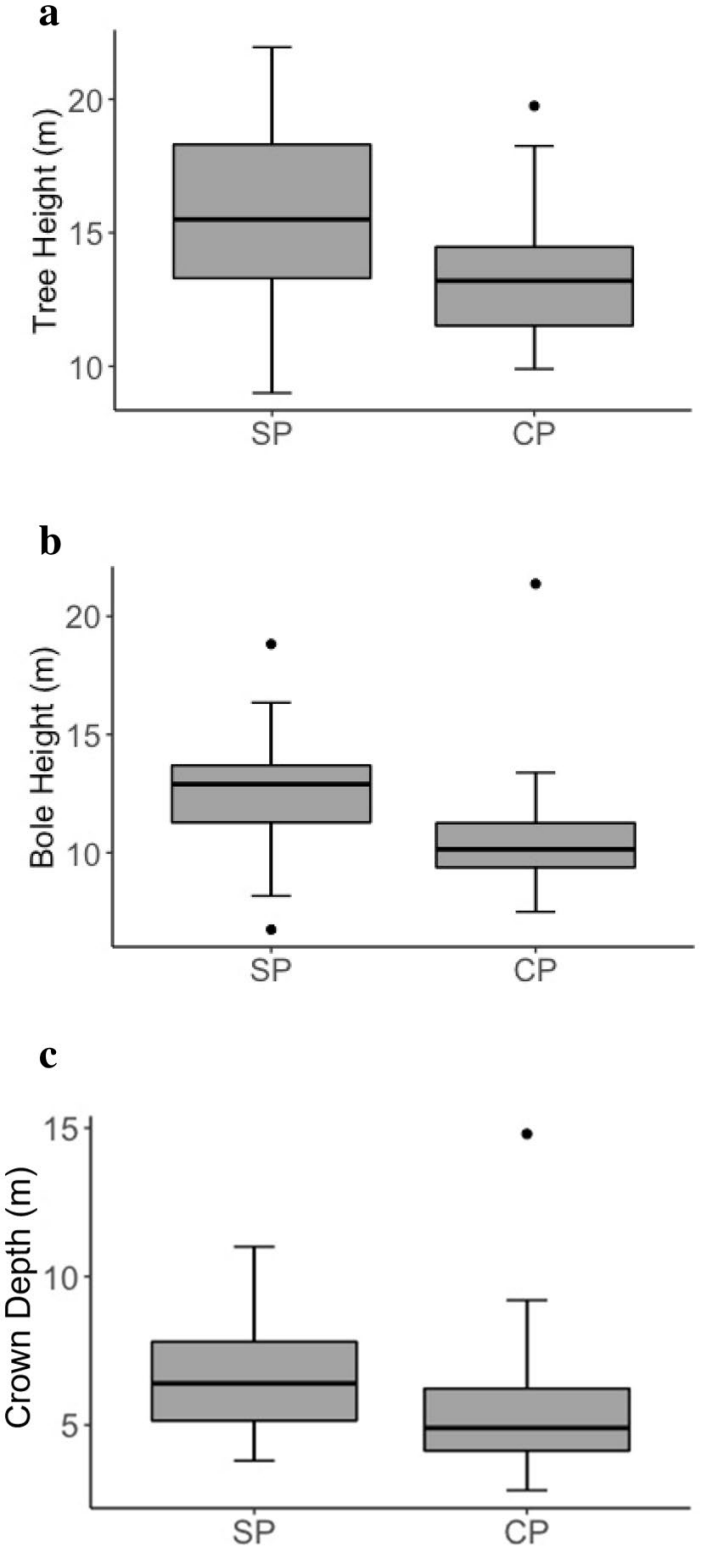
Tree height (**a**), bole height (**b**) and crown depth (**c**) of background trees. *SP *Sleeping plots, *CP *control plots. *Boxes* represent quartiles,* whiskers* are set to the 95th percentile,* dots* represent outliers

Sleeping trees across both siamang units had significantly higher canopy connectivity values (*U* = 86, *P* < 0.001; Fig. 2a), and significantly fewer branches over 20 cm in diameter (*U* = 379, *P* = 0.035; Fig. 2b) than emergent control trees (full results in Table 4). Background trees in sleeping plots were significantly taller (*U* = 144, *P* = 0.001; Fig. 3a) and had higher first major boles (*U* = 132, *P* = 0.016; Fig. 3b), which resulted in greater crown depths (*U* = 148, *P* = 0.048; Fig. 3c), than background trees in control plots.

**Table 4:** Comparison of siamang sleeping trees (*ST*; *n* = 22) with control trees (*CT*; *n* = 21)

Variable	Group	Median	Inter-quartile range	*U*	*P*
DBH (cm)	ST	111.5	59.9	301.5	0.089
	CT	133.6	81.2		
Tree height (m)	ST	41.2	12.7	209	0.605
	CT	40	9.5		
Bole height (m)	ST	30.9	9.5	271	0.341
	CT	30	7.1		
Crown area (m^2^)	ST	227.4	218.9	225	0.895
	CT	225	134.2		
Crown depth (m)	ST	17.6	11.9	180	0.222
	CT	16.2	12.9		
Canopy connectivity (%)	ST	12	17	86	< 0.001*
	CT	2.5	5		
Vines and Lianas (%)	ST	0	5	263	0.377
	CT	0	26.3		
No. branches 10–20 cm	ST	24	17	291	0.148
	CT	28.5	20.5		
No. branches > 20 cm	ST	9	7	379	< 0.001*
	CT	21	18.8		

* *P* < 0.05

FG slept in trees that were significantly taller than emergent control trees (median = 49.00 and 38.95 m, respectively; *U* = 24, *P* = 0.033), with higher boles (median = 36.95 and 29.21 m, respectively; *U* = 24, *P* = 0.033), larger crown areas (median = 367.57 and 209.45 m^2^, respectively; *U* = 14, *P* = 0.004), greater crown depths (median = 26.1 and 16.9 m, respectively; *U* = 21.5, *P* = 0.023), and more canopy connectivity (median = 20 and 2%, respectively; *U* = 20.5, *P* = 0.017). SF also used sleeping trees with higher canopy connectivity than control trees (median = 10% and 2%, respectively; *U* = 42, *P* = 0.036), and with fewer small branches (median = 17 and 34 branches, respectively; *U* = 146, *P* = 0.001) and large branches (median = 9 and 25 branches, respectively; *U* = 151, *P* < 0.001) (full results in Supplementary materials 2). Sleeping trees did not differ from control trees in DBH for either FG or SF (full results in Supplementary materials 1 and 2), indicating that control trees were a suitable control based on their height and the fact that they were emergent (Hankinson et al. in review). Background trees in sleeping plots for FG had larger crown areas (median = 70.68 and 23.92 m^2^, respectively; *U* = 13, *P* = 0.033) and more large branches compared with background trees in control plots (interquartile range = 0.5 and 0 branches, respectively; *U* = 40, *P* = 0.03; full results in Supplementary materials 3). Background trees in sleeping plots for SF were significantly taller compared with background trees in control plots (median = 15.5 and 13.3 m, respectively; *U* = 39.5, *P* = 0.027), with higher boles (median = 13.2 and 10.31 m, respectively; *U* = 29, *P* = 0.006) and greater crown depths (median = 6.00 and 4.85 m, respectively; *U* = 41, *P* = 0.033) (full results in Supplementary materials 4).

FG’s sleeping trees had significantly larger DBH (median = 159.24 and 95.54 cm, respectively; *U* = 97, *P* = 0.002), larger crown areas (median = 367.57 and 177.81 m^2^, respectively; *U* = 93, *P* = 0.003) and crown depths (median = 26.1 and 15.4 m, respectively; *U* = 83, *P* = 0.032), and more branches 10–20 cm in diameter (median = 37 and 17 branches, respectively; *U* = 88.5, *P* = 0.012) (full results in Supplementary materials 5) than those of SF. FG also selected sleeping trees surrounded by background trees with greater crown areas than those of SF (median = 70.68 and 20.6 m^2^, respectively; *U* = 90, *P* = 0.007; full results in Supplementary materials 6).

### Sleep-related behaviours

**Fig. 4:**
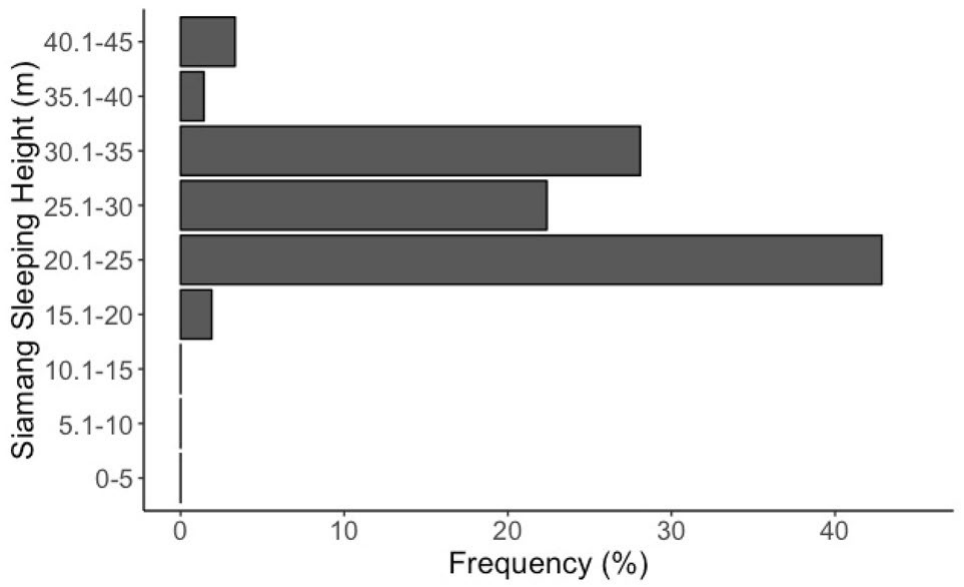
Frequency distribution of siamang sleeping heights (measured from the ground to the sleeping place)

Siamangs always entered sleeping trees before sunset (100%, *n* = 107) and generally left them before sunrise (89%, *n* = 75). They entered sleeping trees 86 ± 60 min before sunset (*n* = 107) and reached their sleeping position within the sleeping tree 5–20 min later (*n* = 99). The first siamang movements (branches moving, short vocalisations) were heard 25 ± 9 min before sunrise (*n* = 57), with siamangs leaving the sleeping trees 15 ± 9 min later (*n* = 75). Siamangs mostly slept at the end of branches (83%, *n* = 173), occasionally at the mid point of a branch (15%, *n* = 32) and rarely near the trunk (2%, *n* = 4). On average, siamangs slept at a height of 25.3 m (median; interquartile range = 21.2 m and 33.0 m; *n* = 210; Fig. 4), 14.0 m (median; interquartile range = 6.7 m and 17.5 m; *n* = 210) from the top of the tree; 9.0 m (median; interquartile range = − 3.5 m and 14.0 m) above the first major bole (*n* = 210); and 16.7 m (median; interquartile range = 9.4 m and 19.0 m;* n* = 210) above the mean canopy height (mean of all background tree heights per plot). Members of FG always slept together in the same sleeping tree, with the adult and sub-adult males often sleeping in an embrace position or less than 1 m apart (86%, *n* = 51), with the adult female sleeping on a separate branch. FG moved from their sleeping tree during the night on three out of 24 observations (determined when we returned to FG’s previous night’s sleeping tree the next morning); on two of these occasions there had been a storm and heavy rainfall. SF moved from her sleeping tree during the night once in 17 observations (inferred in the same way as for FG).

### Home ranges and locations of sleeping trees

**Fig. 5:**
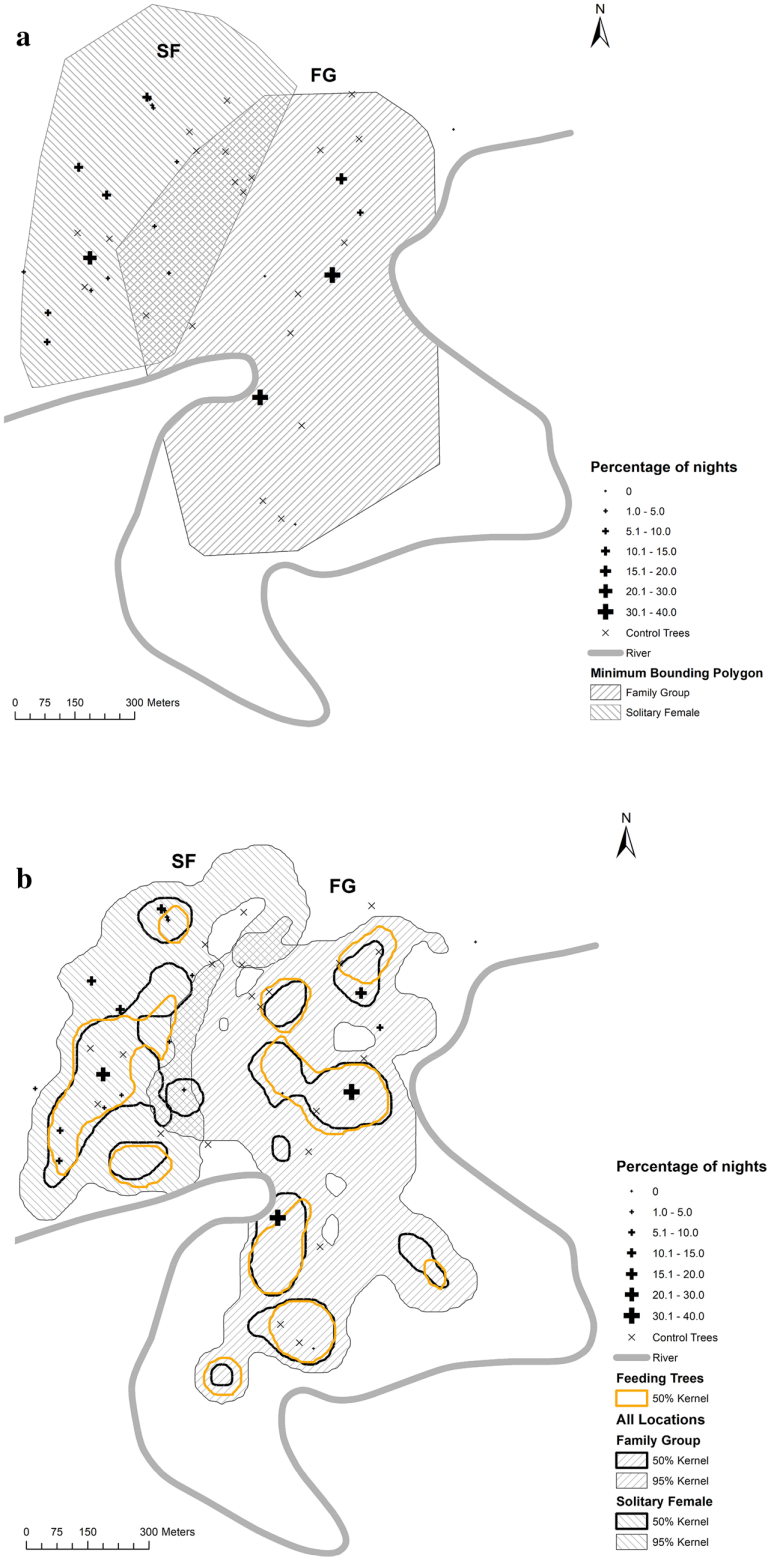
Location of sleeping trees and control trees within the home ranges of both siamang units (SF and FG) calculated using minimum convex polygon (**a**), and the kernel density estimate (KDE;* Kernel*) method to subdivide the core (50% KDE) and periphery (95% KDE) areas of the home range, and the core (50% KDE) areas of feeding trees (**b**). For other abbreviations, see Fig. 1

A total of 1015 GPS waypoints were collected across the two siamang units from April to August 2018 (FG, *n* = 580; SF, *n* = 436). The MCP-defined home range was 0.70 km^2^ for FG and 0.44 km^2^ for SF, with an overlap of 0.12 km^2^ (Fig. 5a), i.e. 17.1% of the home range of FG and 27.3% of the home range of SF, respectively. Sleeping trees were located throughout the two home ranges, but for SF the sleeping trees were notably more widely scattered, including boundary areas and the area of overlap with the FG home range. In contrast, the four sleeping trees used by FG were towards the core of their home range (Fig. 5a). When the home ranges were defined using the KDE, only one of SF’s sleeping trees was in the overlap area based on the 95% KDE (Fig. 5b). Sleeping trees within the core ranges (50% KDE) were used on 91.67% of the nights by FG and 65.71% of the nights by SF (5. 3B). Control plots (*n* = 21) were distributed across both home ranges, with 11 plots exclusively in FG’s home range, five exclusively in SF’s home range, and six in the overlapping area (Fig. 5a). Scramble competition (i.e. for resources that are available to all competitors) with other diurnal primates was recorded when an unstudied and unhabituated solitary female siamang, a group of Thomas’ langurs and a group of pig-tailed macaques were recorded in known sleeping trees of FG on one occasion each per species. One of SF’s sleeping trees was also used once for sleeping in by an unstudied and unhabituated solitary female lar gibbon. On these occasions the studied siamang units slept in other trees. We regarded this to be indirect scramble competition as there were no agonistic encounters for sleeping trees that are considered a limited resource for primates in the area.

### Distances between sleeping trees and feeding trees

The siamangs in this study had a diet that comprised 56% fruit, 42% leaves and 2% other dietary sources (insects, flowers). Sleeping trees were located at a mean distance of 321 ± 365 m (range = 0–1811 m, *n* = 33) from the first feeding tree. The mean distance from the last feeding tree to the sleeping tree was 126 ± 193 m (range = 0–1005 m, *n* = 33). A pairwise comparison indicated that sleeping trees were significantly closer to the last feeding tree than to the first feeding tree (Wilcoxon signed rank test: *z* = − 2.99, *P* = 0.003, *n* = 66). As the siamangs had a daily travel distance of 1.57 km ± 0.3 km (range = 1.1–2.5 km, *n* = 33), the first feeding trees were at an average distance of 20% of their daily travel distance from the sleeping tree. This contrasts with evening data where sleeping trees were at an average distance of 8% of their daily travel distance away from the last feeding tree. One sleeping tree used by FG on 36.67% of nights and seven used by SF on 48.57% of nights were within an intensively used area (50% KDE) of feeding trees. All sleeping trees used by FG and SF were within 100 m of the 50% KDE of feeding trees (Fig. 5b). The mean distances from sleeping trees to the most intensively used areas (50% KDE) of feeding trees were 32.69 m and 24.25 m for FG and SF, respectively.

## Discussion

Our results indicate that siamangs at Sikundur selected sleeping trees based on predator avoidance and distance to feeding sources, with groups of siamangs choosing larger, more stable sleeping trees. Range defence did not influence sleeping tree choice but both siamang units showed scramble competition with other primate species or with other siamangs. Siamangs chose trees that had reduced accessibility, and behaved in ways that decreased the risk of detection and capture by predators. They reused the same sleeping trees frequently, and occasionally on consecutive nights, which increased predictability but could be associated with familiarity with escape routes. Siamangs generally avoided sleeping trees at home range boundaries, suggesting less confrontational range defence but more avoidance of neighbouring conspecifics. Sleeping trees were often close to the last feeding trees, suggesting distance to food resources influences sleeping tree selection. The studied siamangs shared their sleeping sites with other medium-bodied primates, including other unstudied siamangs; further data are required on possible competition for this resource. Finally, members of FG slept in larger, more stable trees than SF, which supports the prediction that a group would select trees based on a group’s requirements.

Siamangs slept in tall trees with wide trunks and large crown areas that emerged above the surrounding canopy, as do other gibbon species (Cheyne et al. 2012; Fan and Jiang 2008; Fei et al. 2012, 2017; Phoonjampa et al. 2010; Reichard 1998; Whitten 1982). The canopy connectivity of emergent control trees was considerably lower than that of sleeping trees, indicating siamangs’ need for physical connections between trees to reach their desired sleeping locations via climbing or brachiation (Fleagle 1976). Nonetheless, connectivity of sleeping trees was still low, which minimised accessibility for predators. Predator access routes can also influence sleeping tree selection (Cheyne et al. 2012). A greater number of larger, more stable branches allows predators to enter a tree, and we observed that sleeping trees had fewer larger branches. Sleeping trees were also surrounded by tall trees with high boles that are difficult for terrestrial predators to reach from the ground. Surrounding tall trees may also create effective escape routes through canopy connectivity, as inferred for black crested gibbons (*Nomascus concolor jingdongensis*) in China (Fan and Jiang 2008).

Siamangs always entered sleeping trees before sunset, similar to agile gibbons (Cheyne et al. 2012) and Cao Vit gibbons (Fei et al. 2012); this is thought to be a strategy for the avoidance of nocturnal predators (Fan and Jiang 2008; Phoonjampa et al. 2010). Clouded leopards are known predators of siamangs (Morino 2010), and were seen on camera traps in this study area. Sleeping at the end of a branch is common in larger primates, including siamangs (Chivers 1974). Predation risk is thought to be reduced by this strategy as it increases the chance of a primate detecting a predator through the vibrations of smaller branches, and because the end of a sleeping branch is less likely to support the combined weight of a primate and a predator (Jay 1956). However, sleeping at the end of branches or on the highest branches could expose primates to avian predators, although siamangs react less to raptors than smaller hylobatids do (Morino 2010). The adult and sub-adult males of FG often slept close to one another or in an embrace, in accordance with other siamang studies (Lappan 2008); this may have helped the smaller juvenile to keep warm. Females carrying infants may be more vulnerable to predators than other individuals because of their reduced speed and agility whilst moving through the canopy. Female lar gibbons with infants chose taller, safer sites for sleeping in (Reichard 1998). In this study, the female siamang of FG always slept away from the two males, but we do not have enough data to draw conclusions about why she did this. Thermoregulatory considerations are unlikely to influence choice of sleeping locations at Sikundur as the average overnight temperature does not drop below 25 °C (Marsh 2019).

Hylobatids in previous studies used many sleeping trees, infrequently used the same tree more than once, and rarely or never on consecutive nights (Fei et al. 2012, 2017; Reichard 1998). Using a sleeping tree repeatedly increases predictability to predators and possibly the risk of disease due to a build-up of faeces. Sleeping tree reuse at our site might reflect a lack of suitable trees with preferred physical characteristics (height, crown area, canopy connectivity, etc.), as reported in bonnet macaques (*Macaca radiata*) (Ramakrishnan and Coss 2001). However, as siamangs appear to use fewer sleeping trees than other gibbon species elsewhere (Morino 2010), this is unlikely to be the sole driving factor. From the prey’s perspective, familiar sleeping sites offer known escape routes should they be attacked (Di Bitetti et al. 2000; Struhsaker 1967). Another possible advantage of having a few sleeping sites that are regularly reused is that individuals who accidentally become separated from the group can rejoin it in the evenings (Ramakrishnan and Coss 2001). Considering how cohesive siamang groups are (Palombit 1996), this may provide an explanation for the very low numbers of sleeping trees used by the FG compared to the SF.

Home ranges are generally larger when the densities of resources such as food and sleeping sites are low (Börger et al. 2008). Siamangs at Sikundur had larger home ranges (0.70 km^2^ for FG and 0.44 km^2^ for SF) than those in unlogged forest at Bukit Barisan Selatan National Park, southern Sumatra (0.20 km^2^) (O'Brien et al. 2003). Siamangs in our study showed a preference for hardwood trees; however, these are targeted during selective logging, which reduces the number of potential sleeping sites for siamangs. At Sikundur, trees of the family Euphorbiaceae had the highest species richness, and species of the family Dipterocarpaceae were most abundant (Priatna et al. 2006); trees of these families accounted for around 30% of the siamang sleeping trees. Life in a group demands greater resources, and FG required bigger, more stable trees with larger crowns and more small branches to support their numbers. This, coupled with the accessibility of the humans, which is at the national park boundary, could have contributed to the low availability of suitable trees and the number of sleeping trees used.

Sleeping trees were distributed throughout the siamangs’ home ranges, with most in the central areas, few at the edges, and two in the overlap area of FG and SF. Selecting trees at the edge of the home range could be an adaptation for range defence, but could also give rise to aggressive territorial encounters, which in gibbons and siamangs can be fatal (Palombit 1993). Although some primate species share sleeping sites (Aquino and Encarnación 1986; Puertas et al. 1995; Radespiel et al. 1998), gibbons and siamangs may be in competition where suitable trees are a finite resource (Anderson 1984). Both intraspecific competition and interspecific competition with medium-sized primates was observed, although this was indirect, and only occurred when the focal siamangs slept in another of their known sleeping trees.

Primates that have multiple sleeping sites that are widely distributed throughout their home range can optimise their time budgets in relation to feeding (Caselli et al. 2017) if they know the optimal routes between resource patches (Börger et al. 2008). Forest primates often move to sleeping sites that are close to their last feeding patch (Anderson 1984); our results agree with this finding, and with animal space use and movement theories (Börger et al. 2008). In fact, on several evenings, the members of FG fed on a fruiting liana that was growing on a sleeping tree. Studies on agile gibbons and lar gibbons, however, failed to find a link between sleeping trees and feeding trees, probably because food is never too far from a sleeping site (Gittins 1982; Reichard 1998). In our study, the siamangs’ diet consisted of 56% fruit, 42% leaves and 2% other food items, compared with 61% fruit and 17% leaves for siamangs at Ketambe (Palombit 1997), and 36% fruit, 48% leaves for siamangs on the Malay peninsula (Gittins and Raemaekers 1980). Temporal and spatial variation in fruiting trees might explain why FG did not use three of their known sleeping trees during the study period.

Our study provides new information on siamang sleeping tree choice and sleep-related behaviours. The physical characteristics of sleeping and surrounding trees, siamang behaviours, sleeping tree locations and group vs. solitary preferences were all shown to be influencing factors. Further research on siamang habitat use and predation pressures would be helpful to further clarify the behavioural ecology of this endangered species. As pressures mount on Sumatra’s forests, their primate populations, including those of siamangs, are expected to continue to decline, and thus would benefit from protection measures designed to help conserve these fragile ecosystems.

## Electronic supplementary material

Below is the link to the electronic supplementary material.

Supplementary file1 (DOCX 141 kb)
