# Supplementary material for: Sleeping trees and sleep-related behaviours of the siamang (Symphalangus syndactylus) in a tropical lowland rainforest, Sumatra, Indonesia
Source: Primates. 2020 Jul 27;62(1):63–75. doi: 10.1007/s10329-020-00849-8 (PMC7813730; doi:10.1007/s10329-020-00849-8)
Supplement: Supplementary file 1 — Supplementary file1 (DOCX 141 kb) [file 10329_2020_849_MOESM1_ESM.docx]

**Supplementary material 1.** Comparing FG sleeping trees (ST, *N* = 7) with control trees (CT, *N* = 16) within their respective home range. Significance level of < 0.05 are indicated by *.

| **Variable** | **Group** | **Median** | **IQR** | ***U*** | ***P*** |
| --- | --- | --- | --- | --- | --- |
| ***DBH (cm)*** | ST | 159.24 | 25.8 | 51 | 0.764 |
|  | CT | 158.76 | 98.96 |  |  |
| ***Tree Height (m)*** | ST | 49 | 12.9 | 24 | 0.033* |
|  | CT | 38.95 | 10.88 |  |  |
| ***Bole Height (m)*** | ST | 36.75 | 9.68 | 24 | 0.033* |
|  | CT | 29.21 | 8.16 |  |  |
| ***Crown Area (m^2^)*** | ST | 367.57 | 109.35 | 14 | 0.004* |
|  | CT | 209.45 | 139.28 |  |  |
| ***Crown Depth (m)*** | ST | 26.1 | 14.45 | 21.5 | 0.023* |
|  | CT | 16.9 | 13 |  |  |
| ***Canopy Connectivity (%)*** | ST | 20 | 12.5 | 20.5 | 0.017* |
|  | CT | 2 | 5 |  |  |
| ***Vines and Lianas (%)*** | ST | 0 | 10 | 59 | 0.853 |
|  | CT | 0 | 18.75 |  |  |
| ***No. Branches 10-20cm*** | ST | 37 | 12.5 | 38.5 | 0.256 |
|  | CT | 23.5 | 27.5 |  |  |
| ***No. Branches >20cm*** | ST | 11 | 16.5 | 74.5 | 0.228 |
|  | CT | 21 | 13.25 |  |  |

**Supplementary material 2.** Comparing SF sleeping trees (ST, *N* = 15) with control trees (CT, *N* = 11) within her respective home range. Significance level of < 0.05 are indicated by *.

| **Variable** | **Group** | **Median** | **IQR** | ***U*** | ***P*** |
| --- | --- | --- | --- | --- | --- |
| ***DBH (cm)*** | ST | 95.54 | 36.62 | 112.5 | 0.126 |
|  | CT | 114.33 | 62.1 |  |  |
| ***Tree Height (m)*** | ST | 41.2 | 10.05 | 73.5 | 0.659 |
|  | CT | 39.6 | 9.8 |  |  |
| ***Bole Height (m)*** | ST | 30.9 | 7.54 | 73.5 | 0.659 |
|  | CT | 29.7 | 7.35 |  |  |
| ***Crown Area (m^2^)*** | ST | 177.81 | 101.55 | 94 | 0.574 |
|  | CT | 215.9 | 65 |  |  |
| ***Crown Depth (m)*** | ST | 15.4 | 6.7 | 69.5 | 0.516 |
|  | CT | 11.1 | 9.95 |  |  |
| ***Canopy Connectivity (%)*** | ST | 10 | 11 | 42 | 0.036* |
|  | CT | 2 | 7.5 |  |  |
| ***Vines and Lianas (%)*** | ST | 0 | 2.5 | 89 | 0.69 |
|  | CT | 0 | 32.5 |  |  |
| ***No. Branches 10-20cm*** | ST | 17 | 12 | 146 | 0.001* |
|  | CT | 34 | 14 |  |  |
| ***No. Branches >20cm*** | ST | 9 | 6 | 151 | <0.001* |
|  | CT | 25 | 15 |  |  |

**Supplementary material 3.** Comparing FG background trees in sleeping plots (SP, *N* = 7) with background trees in control plots (CP, *N* = 16) within their respective home range. Significance level of < 0.05 are indicated by *.

| **Variable** | **Group** | **Median** | **IQR** | ***U*** | ***P*** |
| --- | --- | --- | --- | --- | --- |
| ***DBH (cm)*** | SP | 31.85 | 19.11 | 36 | 0.192 |
|  | CP | 21.42 | 6.29 |  |  |
| ***Tree Height (m)*** | SP | 14.5 | 4.43 | 52.5 | 0.841 |
|  | CP | 13 | 3.49 |  |  |
| ***Bole Height (m)*** | SP | 11.33 | 3.9 | 56.5 | 1 |
|  | CP | 10.09 | 2.55 |  |  |
| ***Crown Area (m^2^)*** | SP | 70.68 | 30.9 | 13 | 0.003* |
|  | CP | 23.92 | 17.77 |  |  |
| ***Crown Depth (m)*** | SP | 6.4 | 2.25 | 45.5 | 0.504 |
|  | CP | 5.03 | 2.24 |  |  |
| ***Canopy Connectivity (%)*** | SP | 50 | 23.75 | 67.5 | 0.461 |
|  | CP | 63.75 | 26.25 |  |  |
| ***Vines and Lianas (%)*** | SP | 5 | 7.5 | 46.5 | 0.514 |
|  | CP | 0 | 5 |  |  |
| ***No. Branches 10-20cm*** | SP | 2 | 1.75 | 36.5 | 0.19 |
|  | CP | 1 | 2 |  |  |
| ***No. Branches >20cm*** | SP | 0 | 0.5 | 40 | 0.03* |
|  | CP | 0 | 0 |  |  |

**Supplementary material 4.** Comparing SF background trees in sleeping plots (SP, *N* = 15) with background trees in control plots (CP, *N* = 11) within her respective home range. Significance level of < 0.05 are indicated by *.

| **Variable** | **Group** | **Median** | **IQR** | ***U*** | ***P*** |
| --- | --- | --- | --- | --- | --- |
| ***DBH (cm)*** | SP | 22.29 | 6.53 | 74 | 0.678 |
|  | CP | 21.18 | 5.1 |  |  |
| ***Tree Height (m)*** | SP | 15.5 | 5.4 | 39.5 | 0.027* |
|  | CP | 13.3 | 1.68 |  |  |
| ***Bole Height (m)*** | SP | 13.2 | 3.02 | 29 | 0.006* |
|  | CP | 10.31 | 1.65 |  |  |
| ***Crown Area (m^2^)*** | SP | 20.6 | 15.72 | 54 | 0.148 |
|  | CP | 23.38 | 10.11 |  |  |
| ***Crown Depth (m)*** | SP | 6 | 2.48 | 41 | 0.033* |
|  | CP | 4.85 | 1.45 |  |  |
| ***Canopy Connectivity (%)*** | SP | 70 | 40 | 76 | 0.755 |
|  | CP | 67.5 | 13.75 |  |  |
| ***Vines and Lianas (%)*** | SP | 0 | 5 | 81 | 0.952 |
|  | CP | 0 | 3.75 |  |  |
| ***No. Branches 10-20cm*** | SP | 2 | 1 | 49 | 0.076 |
|  | CP | 0.5 | 1.5 |  |  |
| ***No. Branches >20cm*** | SP | 0 | 0 | 71.5 | 0.238 |
|  | CP | 0 | 0 |  |  |

**Supplementary material 5.** Comparing FG sleeping trees (*N* = 7) with SF sleeping trees (CT, *N* = 15). Significance level of < 0.05 are indicated by *.

| **Variable** | **Group** | **Median** | **IQR** | ***U*** | ***P*** |
| --- | --- | --- | --- | --- | --- |
| ***DBH (cm)*** | FG | 159.24 | 25.8 | 97 | 0.002* |
|  | SF | 95.54 | 36.62 |  |  |
| ***Tree Height (m)*** | FG | 49 | 12.9 | 74 | 0.139 |
|  | SF | 41.2 | 10.05 |  |  |
| ***Bole Height (m)*** | FG | 36.75 | 9.68 | 74 | 0.139 |
|  | SF | 30.9 | 7.54 |  |  |
| ***Crown Area (m^2^)*** | FG | 367.57 | 109.35 | 93 | 0.003* |
|  | SF | 177.81 | 101.55 |  |  |
| ***Crown Depth (m)*** | FG | 26.1 | 14.45 | 83 | 0.032* |
|  | SF | 15.4 | 6.7 |  |  |
| ***Canopy Connectivity (%)*** | FG | 20 | 12.5 | 63 | 0.288 |
|  | SF | 10 | 11 |  |  |
| ***Vines and Lianas (%)*** | FG | 0 | 10 | 61.5 | 0.469 |
|  | SF | 0 | 2.5 |  |  |
| ***No. Branches 10-20cm*** | FG | 37 | 12.5 | 88.5 | 0.012* |
|  | SF | 17 | 12 |  |  |
| ***No. Branches >20cm*** | FG | 11 | 16.5 | 62.5 | 0.5 |
|  | SF | 9 | 6 |  |  |

| **Variable** | **Group** | **Median** | **IQR** | ***U*** | ***P*** |
| --- | --- | --- | --- | --- | --- |
| ***DBH (cm)*** | FG | 31.85 | 19.11 | 68.5 | 0.273 |
|  | SF | 22.29 | 6.53 |  |  |
| ***Tree Height (m)*** | FG | 14.5 | 4.43 | 35 | 0.237 |
|  | SF | 15.5 | 5.4 |  |  |
| ***Bole Height (m)*** | FG | 11.33 | 3.9 | 26 | 0.659 |
|  | SF | 13.2 | 3.02 |  |  |
| ***Crown Area (m^2^)*** | FG | 70.68 | 30.9 | 90 | 0.007* |
|  | SF | 20.6 | 15.72 |  |  |
| ***Crown Depth (m)*** | FG | 6.4 | 2.25 | 41 | 0.438 |
|  | SF | 6 | 2.48 |  |  |
| ***Canopy Connectivity (%)*** | FG | 50 | 23.75 | 33.5 | 0.191 |
|  | SF | 70 | 40 |  |  |
| ***Vines and Lianas (%)*** | FG | 5 | 7.5 | 68.5 | 0.224 |
|  | SF | 0 | 5 |  |  |
| ***No. Branches 10-20cm*** | FG | 2 | 1.75 | 54.5 | 0.913 |
|  | SF | 2 | 1 |  |  |
| ***No. Branches >20cm*** | FG | 0 | 0.5 | 58.5 | 0.565 |
|  | SF | 0 | 0 |  |  |

**Supplementary material 6.** Comparing FG background trees in sleeping plots (*N* = 7) with SF background trees in sleeping plots (*N* = 15). Significance level of < 0.05 are indicated by *.
